# The Nervo-Vascular System

**Published:** 1889-11

**Authors:** 


**The Nervo-Vascular System.** Arranged by W. Henry Price and S. Potts Eagleton. Examined and approved by John B. Deaver, M. D., Demonstrator of Anatomy in the University of Pennsylvania. Philadelphia: F. A. Davis, publisher. 1889. Price, fifty cents.

These are three admirably arranged charts for the use of students, to assist in memorizing their anatomical studies.

Part I., The Nerves, gives, first, the number, name, function, superficial origin, foramen of exit, and principal distribution of the cranial nerves; then come the spinal nerves, and the sympathetic nerve, all tabulated in large plain type.

Part II., The Arteries, and Part III., The Veins, are similarly arranged, and all can be framed or otherwise hung upon the wall, to be readily seen and studied at odd moments, or to be examined in connection with other subjects.

